# Risk of contamination of nasal sprays in otolaryngologic practice

**DOI:** 10.1186/1472-6815-7-2

**Published:** 2007-03-13

**Authors:** Erdinc Aydin, Evren Hizal, Babur Akkuzu, Ozlem Azap

**Affiliations:** 1 Departments of Otorhinolaryngology, Baskent University Faculty of Medicine, Ankara, Turkey; 2 Departments of Infectious Diseases, Baskent University Faculty of Medicine, Ankara, Turkey

## Abstract

**Background:**

Reusable nasal-spray devices are frequently used in otolaryngologic examinations, and there is an increasing concern about the risk of cross-contamination from these devices. The aim of our study was to determine, by means of microbiologic analysis, the safety of a positive-displacement or pump-type atomizer after multiple uses.

**Methods:**

A reusable nasal spray bottle, pump, and tips were used in the nasal physical examination of 282 patients admitted to a tertiary otolaryngology clinic. The effectiveness of 2 different methods of prophylaxis against microbiologic contamination (the use of protective punched caps or rinsing the bottle tip with alcohol) was compared with that of a control procedure.

**Results:**

Although there was no statistically significant difference in positive culture rates among the types of nasal spray bottles tested, methicillin-resistant coagulase-negative staphylococci were isolated in 4 of 198 cultures.

**Conclusion:**

Given these findings, we concluded that additional precautions (such as the use of an autoclave between sprays, disposable tips, or disposable devices) are warranted to avoid interpatient cross-contamination from a reusable nasal spray device.

## Background

Topical vasoconstrictive and anesthetic agents that exert a decongestant and/or local anesthetic action on the mucous membranes of the nasal and pharyngeal cavities are routinely used in otolaryngologic practice. To improve the ease of the nasal examination and to make the patient more comfortable, such agents are administered before anterior rhinoscopy, flexible fiber optic or rigid endoscopy, or videolaryngoscopy is performed. Devices that deliver topical vasoconstrictive and anesthetic agents usually convey the medication by either of 2 methods: the Venturi principle or the positive-pressure principle. The risk of contamination of the medication delivery systems as well as the risk of atomizer-associated cross-infection has been investigated, and various results have been reported. Obviously, the administration of an aerosolized agent via a single-use atomizer is a definitive (but costly) solution to that problem. In this study, we assessed the risk of the cross-contamination of multiple-use positive-pressure type nasal sprays and discussed relevant findings in the literature.

## Methods

The study consisted of the microbiologic examination of multiple-dose decongestant nasal spray bottles used to administer an aerosolized agent before the anterior rhinoscopic or fiberoptic endoscopic examination of 282 patients admitted to the Baskent University Ear, Nose, and Throat Clinic during March 2005. The Baskent University Institutional Ethics Committee approved the study protocol (Ref No. KA05/07). Written informed consent was obtained from all subjects.

**Figure 1:**
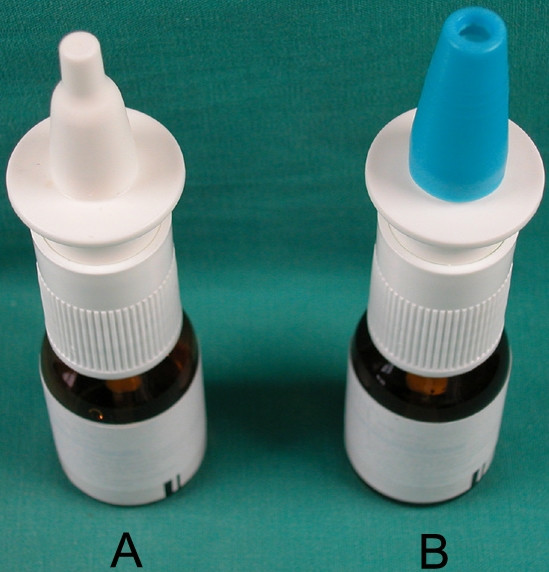
A) A spray bottle with its cap off (group 1). B) A spray bottle with its punched cap on (group 2).

The medication contained in the spray bottles was a combination of 0.05% oxymetazoline hydrochloride (a vasoconstrictive agent) and 0.01% benzalkonium chloride (a preservative) (Iliadin; Merck Ltd, Istanbul, Turkey). The spray bottles were used in 2 different ways. Cap-off bottles (Figure 1) were rinsed with 70% ethyl alcohol solution after each use; cap-on bottles (Figure 1) received a new clean, cap (punched to provide a hole of sufficient size) for each patient.

Each participating physician used 2 spray bottles (1 cap-on bottle and 1 cap-off bottle) on several patients in his or her examination room during the course of single-day outpatient examinations. For each patient requiring nasal decongestion, medication was delivered by a cap-on spray bottle to 1 nostril. A cap-off spray bottle was used to administer medication to the other nostril. During that procedure, the nozzle of the spray bottle was inserted loosely into the nostril, and the bottle was squeezed twice. The patient was asked to "sniff in" to ensure even distribution of the medicated spray. To assess the risk of contamination from the environment, a third and last spray bottle (the unused cap-off control group) was left on the examination table and was not used throughout the day.

At the end of the day, the spray bottles were sent to the microbiology laboratory with the following classifications: cap-off bottles (group 1), cap-on bottles (group 2), or unused cap-off controls (group 3). Each group contained 22 spray bottles, which denoted 22 physician-examination days. Each spray bottle in groups 1 and 2 was used on a mean of 12 ± 2 patients.

**Figure 2:**
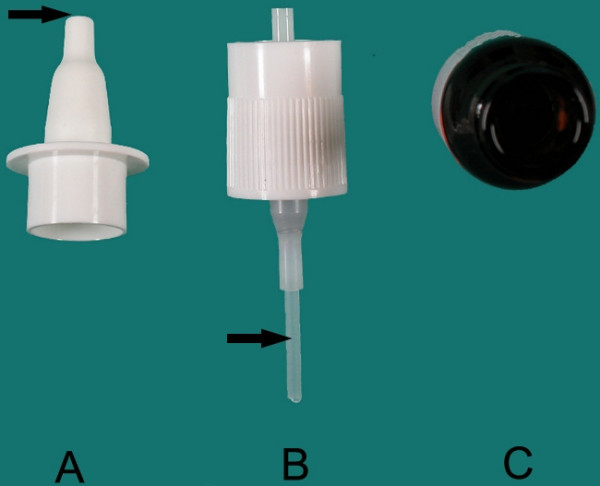
Various parts of the spray bottle that were subjected to culture. A) Tip of the pump. B) Inside of the pump. C) Inside of the spray bottle.

The 3 parts of each spray bottle (the tip, the inner part of the pump, and the fluid remaining in the container) (Figure 2) were applied for culture onto sheep blood, chocolate, and eosin-methylene blue agar. The tip of the pump was streaked onto the agar surfaces. The inside of the pump was rinsed with 1 mL of sterile distilled water, after which 0.01 mL of that fluid was inoculated onto agar surfaces to reveal intraluminal colonization. A sample of the fluid that remained in the spray bottle was also cultured.

The culture plates were incubated for 48 hours. Colonies seen on the agar surfaces were examined, and the bacterial strains were identified by conventional laboratory methods; an additional colony-forming unit count was not performed.

The chi-square test and the Fisher exact test were used for statistical analyses. A *P *value of less than .05 was considered a statistically significant result. Statistical analyses were performed with Epi Info v. 6.0 (Center for Disease Control and Prevention & WHO, Atlanta, Ga, USA).

## Results

Sprays in groups 1 and 2 were used in 282 patients (151 women and 131 men; mean age, 46.2 years; age range, 12–81 years). Each spray was used on an average of 12.8 patients. A total of 198 cultures were performed from 66 spray devices during the study.

Colonies seen on the agar surfaces were further studied, and methicillin-resistant coagulase-negative staphylococcal strains were isolated from all 4 cultures with a positive result. All of the positive results were from samples that had been taken from the tip of the bottles. No bacterial growth was evident in cultures taken from the inner side of the pump or from the fluid that remained in the bottle. Results of the cultures are presented in the Table 1.

**Table 1:** Results of culture from the tip, the inner part of the pump, and fluid remaining in the nasal-spray container

Type of Nasal-Spray Container (group)	Positive Cultures (No.)	Negative Cultures (No.)	Total
1 (Cap-off)	3	63	66
2 (Cap-on)	1	65	66
3 (Control)	0	66	66
Total	4	194	198

There was no statistically significant difference in the positive culture rates among the 3 groups (*P *> .05, x^2 ^= 3.73). In addition, there was no statistically significant difference between groups 1 and 2 when the control group was excluded (*P *> .05, x^2 ^= 0.28).

## Discussion

There are several reports in the literature regarding the risk of contamination of devices that are used to deliver local anesthetics and vasoconstrictive agents to patients in otolaryngology clinics. Atomizers that work via the Venturi principle have been studied; however, the risk of contamination of multiuse sprays that work by means of a positive displacement mechanism has yet to be established [1-10].

In the Venturi effect, the velocity of a gas increases as the corridor through which it passes narrows. The total energy does not change in a closed system, and as the velocity (and therefore the kinetic energy) of the gas increases, its potential energy decreases. The potential energy of a gas is the pressure it exerts; thus, the decrease in potential energy results in a decrease in gas pressure. A Venturi atomizer causes a negative air pressure and creates a vacuum that results in the suction and flow of the fluid out of the medication reservoir and into the medication delivery tube. The fluid column sucked up by the Venturi effect collapses back into the bottle after the airflow has ceased; this results in a transient suction at the tip that can aspirate material into the device. Positive-displacement atomizers do not rely on the Venturi principle but instead use the noncompressible properties of fluid to atomize medications. In positive-displacement atomizers, manually applied kinetic energy is transmitted through a spring-driven pump system to the fluid and causes an increase in the pressure, thus increasing the potential energy of the fluid in the reservoir. The increase in potential energy in turn drives the fluid through the pump lumen and out of the tip. With the aid of a 1-way valve system that prevents the "sucking back" of the fluid and/or a specifically designed air-filter system that prevents the buildup of negative pressure in the bottle, the fluid does not flow back into the bottle. This design theoretically lowers the risk of contamination.

In 1993, Coakley and colleagues reported that nasal sprays that operate via the Venturi principle have the disadvantage of sucking back, which makes them unhygienic for use in more than 1 patient [1]. In a test of that type of atomizer, those authors used a dye that was sucked back into the lumen of the internal nozzle and contaminated the fluid that remained in the reservoir. They concluded that atomizers that work on the Venturi principle must be replaced by disposable atomizers. Wolfe and coworkers used high external concentrations of *Staphylococcus aureus *in solution to investigate the internal contamination of Venturi-principle atomizers and positive-displacement atomizers [2]. They reported that external bacterial contamination of the atomizer nozzle tip resulted in the internal bacterial contamination of the Venturi devices after a single use, but that was not the case in positive displacement devices.

In their study of Venturi atomizers in 12 healthy volunteers, Spraggs and colleagues [3] cultured samples from different parts of the devices and compared the bacterial colonies grown with the colonies from a previously collected nasal swab. The culture results showed the transmission of bacteria from the nasal vestibule onto the nozzle tip, into the nozzle, and into the reservoir of the atomizer and that the preservatives in the medication administered exerted little antibacterial effect.

Southwick and colleagues investigated the association of the transmission of tuberculosis via atomizer reuse and concluded that contaminated atomizers may indeed cause tuberculosis transmission [4]. In contrast, Visosky and colleagues were unable to demonstrate the significant bacterial colonization of multiple-use atomizers in their outpatient otolaryngology clinic [5]. The authors studied serial dilutions of multidose-drug solutions in the atomizers to minimize the inhibitory effect of antiseptic agents in drug formulations and reported that only 0.6% of the drug solutions yielded bacterial growth (coagulase-negative *Staphylococcus*). They concluded that when aerosolizing equipment did not directly contact the patient and the agents that were used contained a bacteriostatic preservative, using multiple-use atomizers did not pose the risk of bacterial transmission among patients. In a study of Venturi-type atomizers containing either lidocaine or tetrahydrozoline hydrochloride (Tyzine) that was applied with the aid of a nasal speculum to reduce contact with the skin and mucosa, Dubin and colleagues [10] showed that wiping the nozzle of the atomizers with an isopropyl alcohol pad after each use resulted in a significant reduction of contamination (from 66% to 6%) in the lidocaine-containing atomizers after 2 weeks, but that after 1 month, no significant difference in the contamination rate between the wiped and nonwiped atomizers was noted. Those authors concluded that although a low level of contamination of multiuse Venturi atomizers might occur in practice, it is of questionable clinical significance and that wiping the tips of the devices with isopropyl alcohol between uses could eliminate microbial growth for a 2-week interval [10]. Similarly, Scianna and colleagues advocate using an appropriate application technique; they stated that continued use of the Venturi-system atomizer is an acceptable practice [6].

In our study, 4 (2.02%) of the 198 cultures taken yielded bacterial growth. In all of the positive cultures, methicillin-resistant coagulase-negative staphylococci were recovered from the tips of the sprayers. The lumen of the pump and the solution that remained in the pump were not contaminated.

Bossart and Wolfe reviewed alternative topical medication applicators that can safely deliver topical medications without the risk of cross-infection [7]. Those authors stated that positive-displacement atomizers with disposable tips can deliver an exact dose of many medications in an atomized spray. Several methods of delivering local anesthetic solutions are preferred in different clinics. Some clinicians use disposable-tip protectors that fit onto the tips of aerosol equipment. The single-use caps that fitted onto the tip of the sprays in group 2 were designed to prevent direct contact with nasal membranes. In our study, we did not necessarily use a nasal speculum to spread the patients' nares; therefore, the tip of the sprays without a cap came into contact with the patients' nasal mucosa. The use of a disposable punched cap seemed to be a practical way of preventing contamination with nasal flora, but 1 of the 4 positive results was obtained from the spray with that type of cap. Although there was no statistically significant difference, there were more positive results of bacterial contamination from sprays without a cap.

Whether the number needed to treat or statistical principles should be used in the evaluation of results like those in our study is a matter of controversy. We suggest that neither of the devices in our study is acceptable.

Because the inner parts of the pump and solutions were not contaminated, replacing the nozzle tip with a new sterilized tip after each use or simply using disposable tips for each patient may be a practical and inexpensive policy. For example, the sterilization of 1 tip with ethylene oxide currently costs about €0.005 at our hospital. However, the safety of that alternative must to be proven in further studies. Furthermore, it is impossible to isolate some important pathogens, such as *Mycobacterium tuberculosis*, viruses, or prions, with conventional microbiologic culture methods (sheep blood, chocolate, and eosin-methylene blue agars). Such pathogens can also easily contaminate the devices and play a role in cross-infection.

## Conclusion

Although no statistically significant difference was present in the groups studied, bacterial growth was identified in 4 of the cultures. Therefore, the ideal method of eliminating cross-infection at the time of this writing is to use a new disposable nasal spray bottle for each patient. We suggest that conducting further studies of this issue with larger groups is necessary, and we concluded that every precaution must be taken to minimize the risk of cross-infection from nasal sprays.

## Competing interests

The author(s) declare that they have no competing interests.

## Authors' contributions

E. A., E. H., and B. A. have contributed to the conception and design of this article and to the analysis and interpretation of data, were involved in drafting and revising the manuscript, have approved the version to be published, and conducted the clinical experiment. O. A. contributed to the conception, design, and drafting and revision of the manuscript and conducted the microbiologic investigations in this study. All authors have read and approved the final manuscript.

## Pre-publication history

The pre-publication history for this paper can be accessed here:


